# The Effects and Molecular Mechanisms of MiR-106a in Multidrug Resistance Reversal in Human Glioma U87/DDP and U251/G Cell Lines

**DOI:** 10.1371/journal.pone.0125473

**Published:** 2015-05-07

**Authors:** Qin Wang, Zhenlian Wang, LinYang Chu, Xu Li, Pengcheng Kan, Xin Xin, Yu Zhu, Ping Yang

**Affiliations:** 1 Department of Clinical Laboratory, Tianjin Huanhu Hospital, Tianjin Key Laboratory of Cerebral Vessels and Neural Degeneration, Tianjin 300060, China; 2 Department of Nursing, School of Pharmaceutical Engineering and Life Science, Changzhou University, Changzhou 213164, China; 3 School of Ophthalmology and Optometry, Eye Hospital, Wenzhou Medical College, Wenzhou 325003, China; 4 The 2nd Department of Orthopedics, the Affiliated Anhui Provincial Hospital of Anhui Medical University, Hefei 230001, China; Swedish Neuroscience Institute, UNITED STATES

## Abstract

Chemotherapy resistance is one of the major obstacles to effective glioma therapy. Currently, the mechanism underlying chemotherapy resistance is unclear. A recent study showed that miR-106a is an important molecule involved in chemotherapy resistance. To explore the effects and mechanisms of miR-106a on multidrug resistance reversal in human glioma cells, we silenced miR-106a expression in the cisplatin-resistant U87 (U87/DDP) and the gefitinib-resistant U251 (U251/G) glioma cell lines and measured the resulting drug sensitivity, cell apoptosis rate and rhodamine 123 content. In addition, we detected decreased expression of P-glycoprotein, MDR1, MRP1, GST-π, CDX2, ERCC1, RhoE, Bcl-2, Survivin and Topo-II, as well as reduced production of IL-6, IL-8 and TGF-β in these cell lines. Furthermore, we found decreased expression of p-AKT and transcriptional activation of NF-κB, Twist, AP-1 and Snail in these cell lines. These results suggest that miR-106a is a promising therapeutic target for the treatment of human multidrug resistant glioma.

## Introduction

Currently, platinum drugs and epidermal growth factor receptor (EGFR) inhibitors are the potential selectable anticancer drugs for the clinical treatment of low grade glioma patient and children patient in China [1–2]. Cisplatin modulates DNA replication, transcription and other cellular processes in tumor cells by forming tight complexes with cellular DNA, leading to anticancer effects including DNA damage and tumor cell apoptosis [3]. Tumor cells resist the cytotoxic effects of cisplatin through a variety of mechanisms, such as increasing drug efflux, repairing DNA and inhibiting apoptotic signals. EGFR is a tyrosine kinase receptor and a member of the epidermal growth factor family. Increased EGFR expression is often detected in glioma. Both deterioration and relapse of glioma are associated with EGFR mutations. Therefore, EGFR is an important target for the clinical treatment of glioma. Gefitinib is an EGFR inhibitor that is widely used in the treatment of glioma carrying a mutation in EGFR [4]. However, in clinical applications of these two drugs, acquired drug resistance often becomes an important issue that affects the patients’ survival time. Hence, it is of great significance to study the mechanism of drug resistance in tumor cells and to develop new methods for drug resistance reversal [5–6]. Previous studies have found that the mechanisms by which tumor cells develop drug resistance include reduced drug absorption, increased drug efflux through the ABC (ATP-binding cassette) transporter protein, enhanced tumor cell ability to detoxify anticancer drugs through the reductase system, reduced tumor apoptosis rate through regulating the apoptosis pathway, and modulation of cytokine production to alter the tumor microenvironment and signaling pathways [7].

Mature microRNAs (miRNA) are a non-coding single-strand small molecule RNAs that are 21–25 nucleotides in length. They have partially complementary sequences to single or multiple messenger RNAs (mRNAs) located at the 3' or 5' ends of RNA precursors. The main function of microRNAs is to down-regulate the expression of genes through mechanisms including translation inhibition, mRNA degradation and deadenylation. MicroRNAs also play important roles in tumor development. Their expression statuses can be used as indicators for tumor diagnosis, classification and prognosis. Increased or decreased microRNA expression has been reported in different types of tumors. MicroRNAs show a significant effect on cell proliferation, survival, apoptosis and the cell cycle [8–9].

MiR-106a is a newly discovered microRNA. Abnormal expression of miR-106a was detected in gastric cancer, colon cancer and esophageal cancer. MiR-106a regulates a variety of functions, such as cell proliferation, tumor invasion and metastasis [10–11]. Although previous studies confirmed an important role for miR-106a in cancer [12], its effect on chemotherapeutic drug resistance in glioma has not yet been reported. This study explored the role of miR-106a in the development of resistance to cisplatin and gefitinib in glioma cells.

## Materials and Methods

### Cell Lines and Cell Culture

The human glioma cell lines U87, U87/DDP, and U251 were purchased from American Type Culture Collection (ATCC) and cultured in Dulbecco’s Modified Eagle Medium (DMEM) containing 10% fetal bovine serum at 37˚C, in 5% CO_2_ with saturated humidity.

The gefitinib-resistant U251 cell line (U251/G) was established by prolonged exposure to chemotherapeutic drug treatment. To verify the drug resistance in U251 cells, cells were cultured in gefitinib-free medium for 4 days. Gefitinib was then added, and MTS was measured. We cultured U251/G cells in drug-free medium for 10 generations and then treated these cells with gefitinib again. Our MTS assay results found that the drug sensitivity of these cells was not restored.

MiR-106a inhibitors and negative miRNA control inhibitors were from RiboBio (China). Cells were transfected with a miR-106a inhibitor or the corresponding control using Lipofectamine RNAiMAX (Invitrogen Life Technologies) according to the manufacturer’s instructions. Quantitative assays to detect miR-106a were carried out using TaqMan microRNA probes (Applied Biosystems) according to the manufacturer’s instructions. There were 8 experimental groups in this study. The parent groups were U87 or U251, the control groups were U87/DDP or U251/G, the miR-106a-NC groups were U87/DDP or U251/G cells transfected with negative miRNA control inhibitors, and the miR-106a-inhibitor groups were U87/DDP or U251/G cells transfected with miR-106a inhibitors.

### MTS assay

Cells (4 × 10^3^) were seeded into 96-well plates and incubated for 24 h. U87 or U87/DDP cells were treated with 0, 0.1, 0.5, 1, 5, 10, 50 or 100 μM cisplatin. U251 or U251/G cells were treated with 0, 0.01, 0.05, 0.1, 0.5, 1, 5, or 10 μM gefitinib. Treated cells were incubated for 72 h. After culturing, 20 μl of MTS (Promega, Madison, WI, USA) was added to each well, and cells were incubated for another 2 h. Absorbance was measured at 492 nm to estimate the tumor cell sensitivity to cisplatin or gefitinib.

### Cell apoptosis assay

Cells (3 × 10^5^) were seeded into 6-well plates and incubated for 24 h at 37˚C, 5% CO_2_ with saturated humidity. U87 or U87/DDP cells were treated with 10 μM cisplatin, and U251 or U251/G cells were treated with 0.1 μM gefitinib for 72 h. The cells were then detached by trypsinization, centrifuged, washed 3 times in phosphate buffered saline (PBS), resuspended in AnnexinV-binding buffer and incubated with AnnexinV- Fluorescein isothiocyanate (FITC) and propidium iodide (PI) for 15 min. Cells were then analyzed by flow cytometry at 488 nm according to the manufacturer’s instructions.

### Drug excretion and retention assays

P-glycoprotein was one of the drug transporters that determine the uptake and efflux of a range of drugs. Tumor cells which overexpressing P-glycoprotein was found reduced the access of cytotoxic drugs. Drug clearance was measured by cell surface P-glycoprotein (P-gp) expression. Cells (3 × 10^5^) were seeded into 6-well plates and incubated for 24 h, detached by trypsinization, centrifuged, washed 3 times in PBS, and incubated with P-gp-PE antibody for 15 min. The cells were then analyzed by flow cytometry at 488 nm according to the manufacturer’s instructions.

Cellular drug retention was measured by intracellular rhodamine 123 (Rh-123) content. Cells (3 × 10^5^) were seeded into 6-well plates and incubated for 24 h, detached by trypsinization, centrifuged, washed 3 times in PBS, and then incubated with Rh-123 for 30 min. The cells were then analyzed by flow cytometry at 488 nm according to the manufacturer’s instructions.

### Liquidchip assay

Cells (3 × 10^5^) were seeded into 6-well plates and incubated for 24 h. Culture medium was replaced with fresh serum-free DMEM, and the cells were incubated for 24 h. The medium was then collected and centrifuged at 1,000 × g for 5 min. The levels of IL-6, IL-8 and TGF-β in the supernatant were measured by a Liquidchip assay (Qiagen, Valencia, CA, USA) according to the manufacturer’s instructions.

### Western blotting assay

Cells (3 × 10^5^) were seeded into 6-well plates and incubated for 24 h, lysed, centrifuged at 4℃ at 14,000 × g for 5 min. The concentration of protein in the supernatant was measured by a Bradford assay. A 100 μg protein sample was separated on a 12% SDS-PAGE gel and transferred onto a PVDF membrane. The membrane was blocked with 5% skim milk for 2 h at room temperature. Monoclonal antibodies against MDR1 (1:800, Santa Cruz), MRP1 (1:800, Santa Cruz), GST-π (1:800, Santa Cruz), CDX2 (1:800, Santa Cruz), Bcl-2 (1:800, Santa Cruz), Survivin (1:800, Santa Cruz), Topo-Ⅱ(1:800, Santa Cruz), p-AKT (1:500, Santa Cruz) and β-actin (1:5000, Sigma) were added and incubated at 4°C overnight in 5% BSA. The membrane was washed 3 times with Tris-buffered saline (TBS) containing 0.1% Tween 20. Horseradish peroxidase (HRP)-labeled secondary antibody (1:1000, Sigma) was added and incubated at room temperature for 1 h. After the last membrane wash, protein was visualized using a DAB kit (Millipore).

### Real-time PCR assay

Cells (3 × 10^5^) were seeded into 6-well plates and incubated for 24 h. Total RNA was extracted using TRIzol Reagent (Life Technologies). Reverse transcription was performed using Superscript VILO (Life Technologies). Quantitative PCR was performed with TaqMan Master Mix (Life Technologies). The following primers were used: MDR1, sense, 5’-ATGTTGAGCCGGGCAGTGTGC-3’ and anti-sense, 5’-GGCTGTTGTCTCCATAGGCAAT-3’; MRP1, sense, 5’- CTTCTGGAGGAATTGGTTGTATAGAAG-3’, and anti-sense, 5’-GGTAGACCCAGACAAGGATGTTAGA-3’; GST-π, sense, 5’-TTCCTTACTGGTCCTCACATCTC-3’and anti-sense, 5’-TCACCGGATCATGGCCAGCA-3’; CDX2, sense, 5’-CTGGAGCTGGAGAAGGAGTTTC-3’and anti-sense, 5’-ATTTTAACCTGCCTCTCAGAGAGC-3’; Bcl-2, sense, 5’-TGCACCTGACGCCCTTCAC-3’and anti-sense, 5’-AGACAGCCAGGAGAAATCAAACAG-3’; Survivin, sense, 5’-GAATTCATGGGTGCCCCGACGTTGCC-3’and anti-sense, 5’-AGATCTTTCTTCTTATTGTTGGTTTCC-3’; Topo-Ⅱ, sense, 5’-TCCTGGGAGTTGGTGTGTCAG-3’and anti-sense, 5’-AAACCCTGCTCGGATCGCTC-3’; GAPDH, sense, 5'-ATGGAAATCCCATCACCATCTT-3' and anti-sense, 5'-CGCCCCACTTGA TTTGG-3'. PCR reaction conditions were as follows: 95°C for 10 min followed by 40 cycles of 95°C for 15 s and 60°C for 60 s.

### Dual-Luciferase Reporter Assay

Cells (3 × 10^4^) were seeded into 24-well plates, incubated for 24 h and then co-transfected with reporter plasmids for NF-κB, Twist, AP-1, and Snail using Lipofectamine 2000 reagent (Invitrogen, USA). Luciferase assays were performed using the Dual-Luciferase Reporter Assay system (Promega, USA) according to the manufacturer’ s instructions. Cytomegalovirus (CMV) was included as an internal reference.

### Statistical analysis

Data were presented as the mean ± SD (standard deviation) and analyzed by SPSS 13.0 software. Statistical analyses were performed using a one-way ANOVA. P < 0.05 was considered significant.

## Results

### MiR-106a reduces the sensitivity of tumor cells to cisplatin and gefitinib

**Fig 1:**
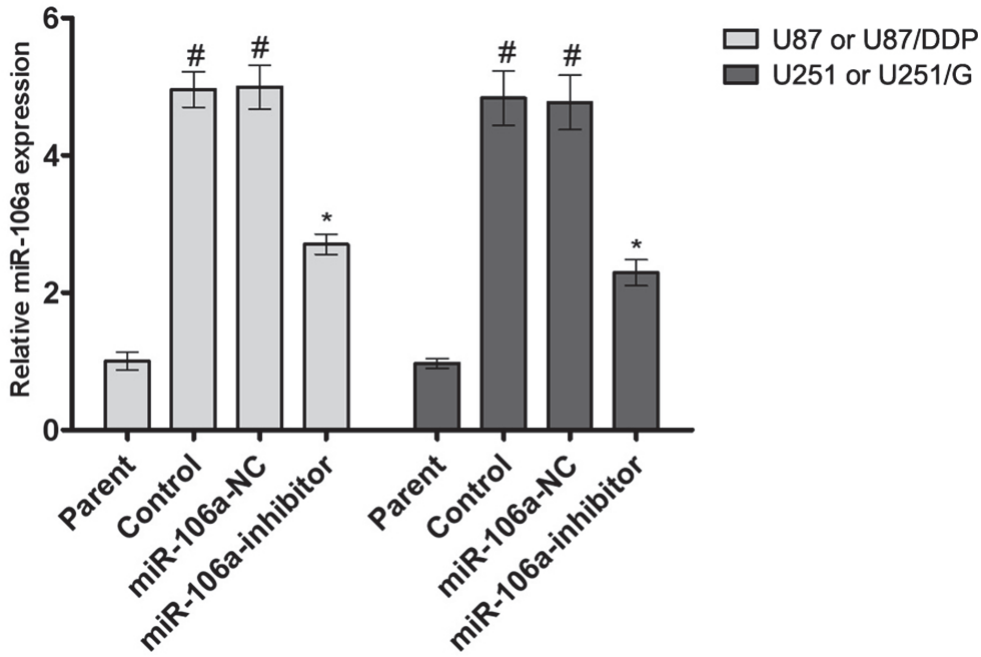
The expression of miR-106a in different human glioma cell lines. The expression of miR-106a in human glioma U87, U87/DDP, U251 and U251/G cell lines. Values are presented as the mean ± SD, n = 5; ^#^compared to the parent group, P < 0.05. *compared to the control group, P < 0.05.

To explore the role of miR-106a in tumor cell drug resistance, we detected the expression of miR-106a in normal U87 cells and the cisplatin-resistant cell line U87/DDP, as well as in normal and gefitinib-resistant U251 cells (U251/G, see Materials and Methodology for construction method). As shown in Fig 1, the realtime PCR assay showed that miR-106a expression was significantly increased in both the cisplatin-resistant cell line and the gefitinib-resistant cell line. Expression of miR-106a was significantly decreased in miR-106a-inhibited glioma cell lines.

**Fig 2:**
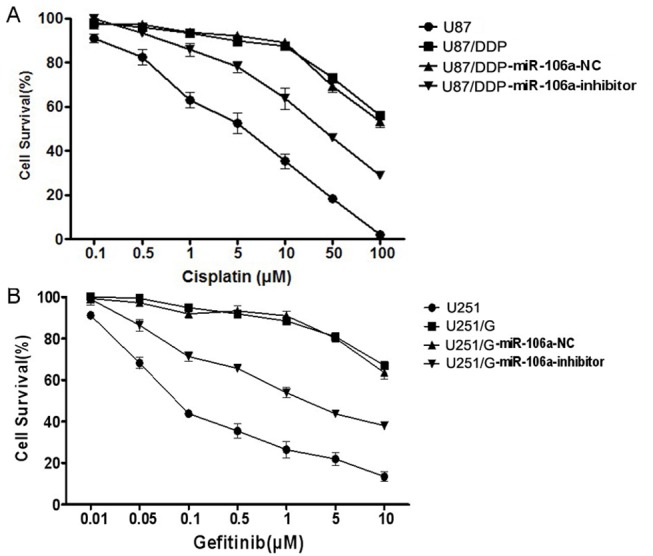
The effect of miR-106a on cisplatin drug sensitivity in human glioma cell lines. (A) The effect of miR-106a on cisplatin drug sensitivity of U87 and U87/DDP cells. Values are presented as the mean ± SD, n = 5. (B) The effect of miR-106a on cisplatin drug sensitivity of U251 and U251/G cells. Values are presented as the mean ± SD, n = 5.

The cisplatin half maximal inhibitory concentration (IC50) in U87/DDP cells was 127.6 μM, which was significantly increased compared with that of U87 cells (IC50 of 5.7 μM). The IC50 of gefitinib in U251 cells was 0.08 μM, a significant increase compared with that of U251/G cells (IC50 of 16.2 μM). The effect of miR-106a on drug sensitivity in glioma cell lines was evaluated. The drug sensitivity of both U87/DDP and U251/G cells was significantly enhanced (48.3 μM and 5.8 μM, respectively) after inhibiting miR-106a (Fig 2). These results indicate that miR-106a plays an important role in promoting the development of drug resistance in tumor cells.

### MiR-106a inhibits chemotherapeutic drug-mediated apoptosis

**Fig 3:**
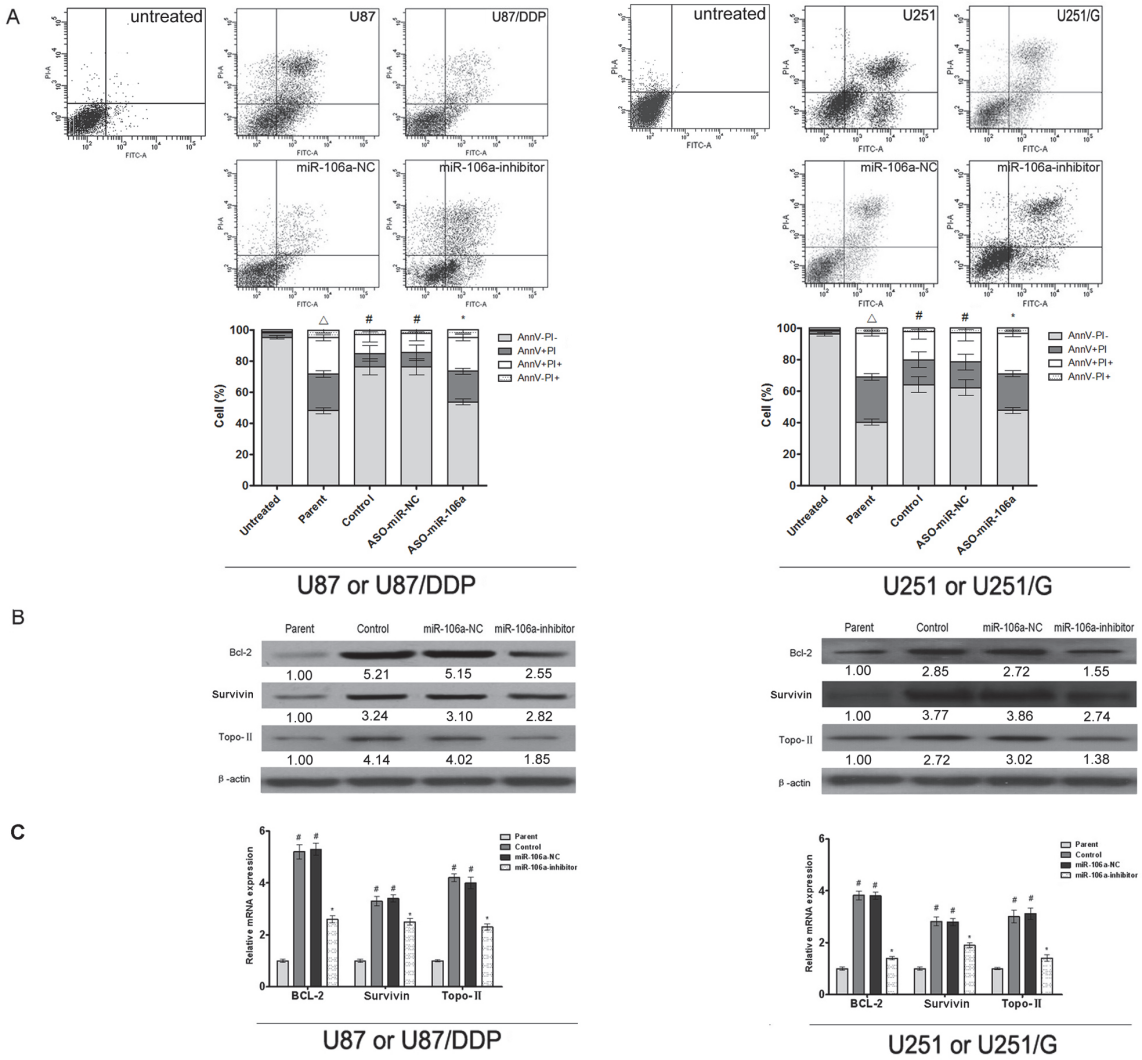
The effect of miR-106a on the apoptosis of human glioma cell lines. (A) The effect of miR-106a on the apoptosis of U87 and U87/DDP or U251 and U251/G cells. Values are presented as the mean ± SD, n = 3. ^△^compared to the untreated group, P < 0.05.^#^compared to the parent group, P < 0.05. *compared to the control group, P < 0.05. (B) The effect of miR-106a on the protein expression of Bcl-2, Survivin and Topo-II in U87 and U87/DDP or U251 and U251/G cells. β-actin was used for normalization. (C) The effect of miR-106a on the mRNA expression of Bcl-2, Survivin and Topo-II in U87 and U87/DDP or U251 and U251/G cells.GAPDH was used for normalization. Values are presented as the mean ± SD, n = 5. ^#^compared to the parent group P < 0.05. *compared to the control group, P < 0.05.

To examine the mechanism of miR-106a-mediated changes on drug sensitivity in tumor cells, we studied the effect of miR-106a on drug-mediated apoptosis. As shown in Fig 3A, the apoptosis rate of miR-106a-inhibited U87/DDP cells was significantly increased compared with that of the control group. Similarly, the apoptosis rate of U251 cells treated with gefitinib was significantly higher than that of the U251/G cells. After miR-106a inhibition, the apoptosis rate of U251/G cells was significantly increased to a level similar to that of the U251 cells.

At the same time, we examined the expression levels of Bcl-2 and Survivin. These two proteins belong to the Bcl-2 family and the inhibitor of apoptosis (IAP) family, respectively, which regulate apoptosis. MiR-106a inhibition significantly decreased the expression of these proteins in both U87/DDP and U251/G cells. Topo-II can also inhibit chemotherapeutic drug-mediated apoptosis (5). MiR-106a inhibition also decreased the expression of Topo-II in both U87/DDP and U251/G cells (Fig 3B).

### MiR-106a promotes drug efflux and enhances cell detoxification and DNA repair by promoting the expression of drug resistance-related genes

**Fig 4:**
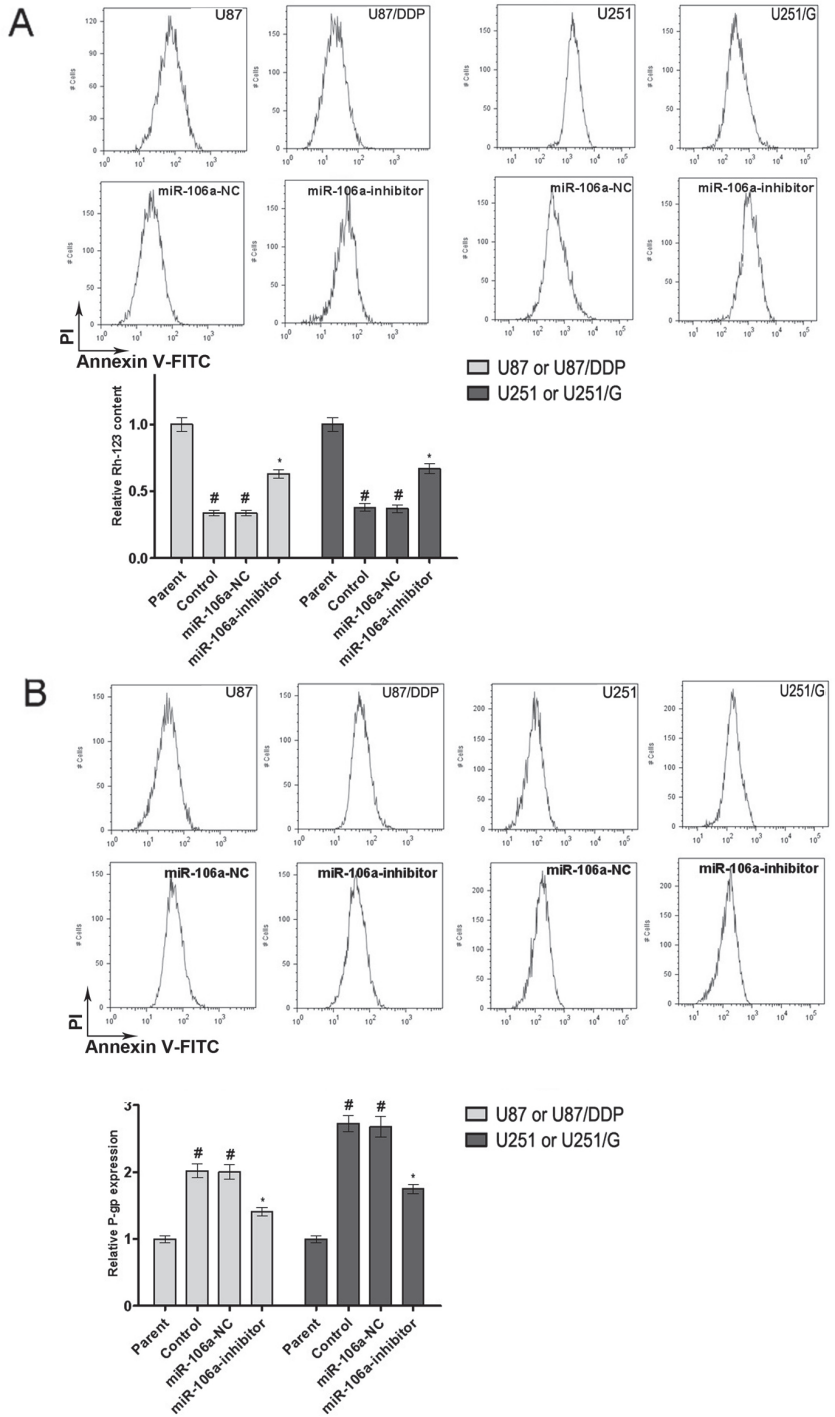
The effect of miR-106a on the drug efflux of human glioma cell lines. (A) The effect of miR-106a on the intra-cellular Rh-123 content in U87 and U87/DDP or U251 and U251/G cells. Values are presented as the mean ± SD, n = 3. ^#^compared to the parent group, P < 0.05. *compared to the control group, P < 0.05. (B) The effect of miR-106a on P-glycoprotein expression in U87 and U87/DDP or U251 and U251/G cells. Values are presented as the mean ± SD, n = 3. ^#^compared to the parent group, P < 0.05. *compared to the control group, P < 0.05.

We hypothesized that miR-106a mediates drug resistance through promoting drug efflux from tumor cells. We therefore examined the main indicators of drug efflux. The efflux capacity of U87/DDP and U251/G cells was detected using rhodamine 123 dye [13]. We found that the intracellular content of rhodamine 123 decreased in both miR-106a-inhibited U87/DDP and U251/G cells, indicating that miR-106a enhances cell efflux. Furthermore, P-gp (P-glycoprotein) was more commonly referred to MDR1, which plays an important role in drug disposition and distribution, the flow cytometry assay result showed that the expression of P-gp decreased on the surface of both groups of miR-106a-inhibited cells (Fig 4).

MDR1 and MRP1 are important genes for the development of multi-drug resistance in tumor cells. In this study, the expression of MDR1 and MRP1 in U87/DDP and U251/G cells were examined. The expression levels of both genes were significantly higher in U87/DDP cells than in U87 cells. Similarly, the expression levels of both genes were also higher in U251/G cells than in U251 cells. However, the expression levels of MDR1 and MRP1 both decreased in miR-106a-inhibited U87/DDP and U251/G cells.

In 2010, Takakura et al. found that CDX2, a member of the caudal-related homeobox transcription factor gene family, can positively regulate the expression of MDR1 [14] and thus modulate the multi-drug resistance of tumor cells. We examined the expression of CDX2 and found that its expression decreased in miR-106a-silenced U87/DDP and U251/G cells.

GST-π is an important cellular detoxification enzyme. Numerous studies have demonstrated that the expression of this protein is increased in drug-resistant tumor cells to counteract the cytotoxicity of anticancer drugs. The expression of GST-π in U87/DDP and U251/G cells was higher than in U87 and U251 cells, respectively, but the expression of GST-π in miR-106a-inhibited U87/DDP and U251/G cells was significantly decreased. The above results indicate that miR-106a may enhance the detoxification capacity of cells by positively regulating GST-π expression, leading to drug resistance.

ERCC1 is an important gene for DNA repair in cells. The development of drug resistance in tumor cells often involves the enhancement of DNA repair capacity ^1^. In glioma, ERCC1 expression is often increased. We found that the expression of ERCC1 in U87/DDP and U251/G cells was significantly higher than that in the control cell lines U87 and U251. We next explored the effect of miR-106a on the expression of ERCC1. We found that inhibiting miR-106a significantly decreased the expression of ERCC1 in both U87/DDP and U251/G cells to the level of the corresponding control group. These results indicate that miR-106a can enhance the DNA repair capacity of the tumor cells.

**Fig 5:**
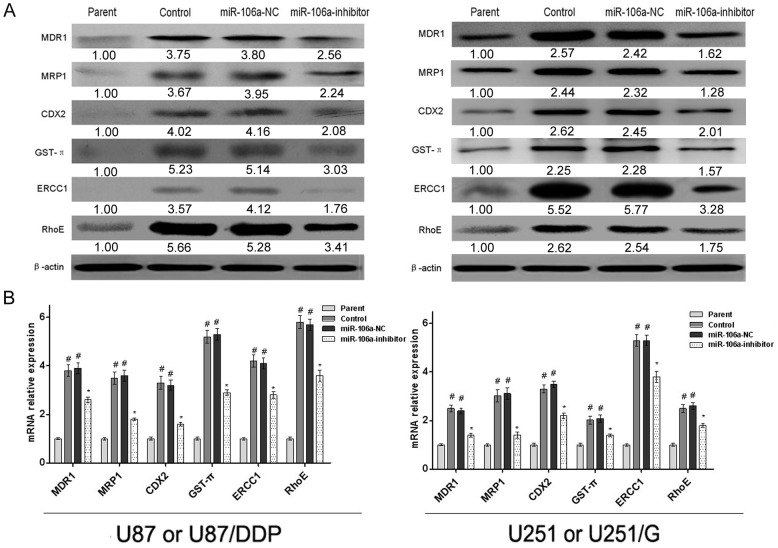
The effect of miR-106a on the expression of drug resistance-related genes in human glioma cell lines. (A) The effect of miR-106a on the protein expression of MDR1, MRP1, CDX2, GST-π, ERCC1 and RhoE in U87 and U87/DDP or U251 and U251/G cells. β-actin was used for normalization. (B) The effect of miR-106a on the mRNA expression of MDR1, MRP1, CDX2, GST-π, ERCC1 and RhoE in U87 and U87/DDP or U251 and U251/G cells. GAPDH was used for normalization. Values are presented as the mean ± SD, n = 5. ^#^compared to the parent group, P<0.05. *compared to the control group, P < 0.05.

RhoE is a member of the Rho protein family. Recent studies have confirmed that this protein is also involved in the development of multi-drug resistance in tumor cells. The expression of RhoE in the control group was significantly lower than that in the drug-resistant cells. In addition, the expression of RhoE decreased after inhibiting miR-106a (Fig 5).

### MiR-106a increases levels of IL-1β, IL-6, IL-8 and TGF-β

**Fig 6:**
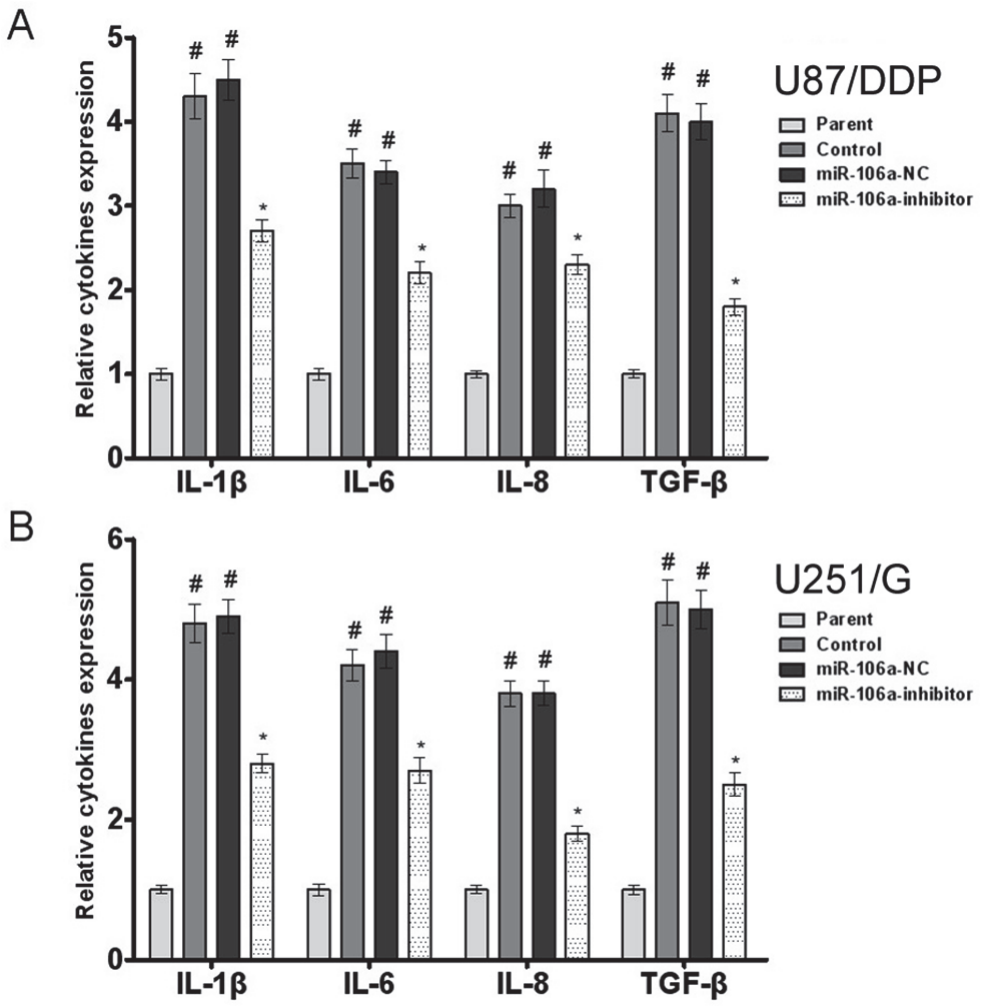
The effect of miR-106a on cytokine production in human glioma cell lines. (A) The effect of miR-106a on the production of IL-6, IL-8 and TGF-β in U87 and U87/DDP cells. Values are presented as the mean ± SD, n = 5. ^#^compared to the parent group, P < 0.05. *compared to the control group, P<0.05. (B) The effect of miR-106a on the production of IL-6, IL-8 and TGF-β in U251 and U251/G cells. Values are presented as the mean ± SD, n = 5. ^#^compared to the parent group, P < 0.05. *compared to the control group, P < 0.05.

Cytokines can affect the tumor microenvironment and signal transduction in tumor cells. The cytokines IL-1β, IL-6, IL-8 and TGF-β play important roles in the development of drug resistance in tumor cells. Our results showed that miR-106a inhibition reduced IL-1β, IL-6, IL-8 and TGF-β production, indicating that miR-106a may contribute to tumor cell drug resistance by promoting the production of these cytokines (Fig 6).

### MiR-106a activates important tumor signaling pathways

**Fig 7:**
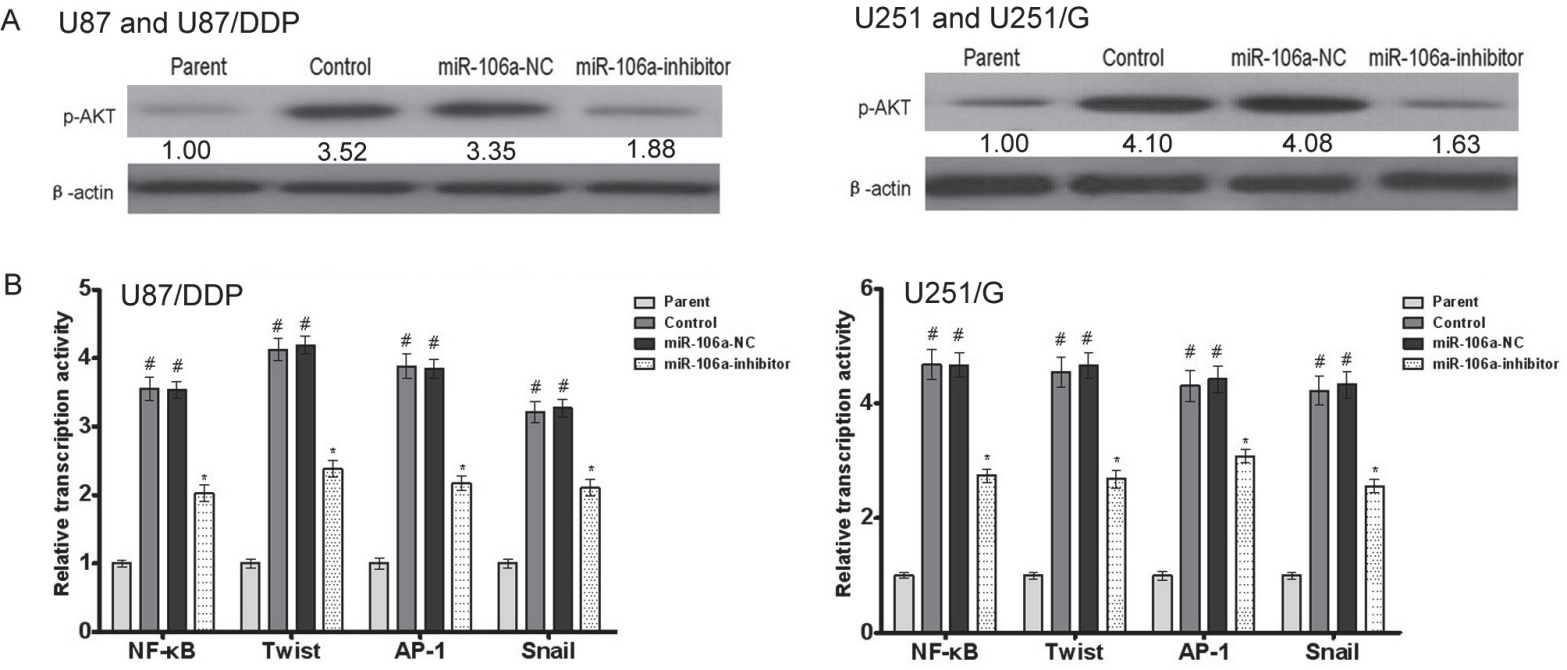
The effect of miR-106a on drug resistance-related signaling molecules in human glioma cell lines. (A) The effect of miR-106a on the phosphorylation of AKT in U87 and A549U87/DDP or U251 and U251/G cells. β-actin was used for normalization. (B) The effect of miR-106a on the phosphorylation of the transcriptional activity of NF- κB, Twist, AP-1 and Snail in U87 and U87/DDP or U251 and U251/G cells. Values are presented as the mean ± SD, n = 5. ^#^compared to the parent group, P < 0.05. *compared to the control group, P < 0.05.

The activation of tumor signaling pathways is one of the most important steps in tumorigenesis. We examined the effect of miR-106a on key proteins in the primary tumor signaling pathway. We first examined AKT activation. In U87/DDP and U251/G cells, p-AKT was significantly higher than in the control group. However, after miR-106a knockdown, the p-AKT level in these two cell lines significantly decreased. Similar trends were observed for important transcription factors. Four transcription factors, NF- κ B, Twist, AP-1 and Snail, were examined in this study. The transcriptional activity of the control group cell lines (U87 and U251) was significantly lower than that of the drug-resistant cell lines (U87/DDP and U251/G). After miR-106a inhibition, the transcription of NF- κB, Twist, AP-1 and Snail all decreased (Fig 7). The results indicate that miR-106a can positively activate tumor cell transcriptional signaling pathways.

## Discussion

With advancing research, the important roles of microRNAs in the initiation and development of tumors have been gradually revealed. MiR-106a is aberrantly expressed in gastric cancer, esophageal cancer, glioma and colorectal cancer [15–17]. MiR-106a may modulate cell proliferation, tumor invasion and metastasis, as well as the cell cycle and EMT [18]. However, the role of miR-106a in tumor development remains controversial.

Cisplatin was a conventional anti-tumor drug with long time clinical application for cancer treatment and was selectable applied in glioma treatment [19]. Especially, the epidermal growth factor receptor (EGFR) was involved in human tumors by regulating important cellular processes and several SNPs in the EGFR gene may be related to glioma risk. As it is known to be amplified and/or mutated in up to 40% of malignant glioma, EGFR associated with glioma in the Chinese population in some study, was one of the key oncogene subjected to targeted therapy for glioma in China [20–21]. Therefore, cisplatin and gefitinib are two potential selectable chemotherapeutic drugs for the clinical treatment of low grade glioma patient and children patient in China.

In this study, the role of miR-106a in drug resistance in glioma was examined. We found that miR-106a enhanced tumor cell resistance to both cisplatin and gefitinib. After miR-106a knockdown, the drug sensitivity of cells to cisplatin and gefitinib significantly increased. Correspondingly, the cell apoptosis rate also increased.

Tumor cell drug resistance involves several mechanisms. MDR1/P-gp and MRP1 are members of the ATP-binding cassette (ABC) transporter superfamily. Increased MDR1/P-gp and MRP1 expression is an important mechanism for the development of multidrug resistance [22]. Specifically, MDR1/P-gp and MRP1 help cells to develop drug resistance by pumping drugs out of the cells and decreasing the intracellular drug concentration. MiR-106a increased the expression of MDR1/P-gp and MRP1 and increased the cell efflux capacity of the tumor cells. CDX2 positively regulates the expression of MDR1/P-gp, and miR-106a also enhanced the expression of CDX2. Thus, miR-106a may upregulate the expression of MDR1/P-gp and MDR1 by promoting CDX2 expression, which in turn enhances the cell efflux capability, leading to drug resistance. We found that miR-106a also increased the expression of RhoE and Topo-II. The role of RhoE [23] and Topo-II [24] in drug resistance in tumor cells has been reported. These proteins counteract the effect of antitumor drugs by inhibiting the apoptosis pathway. Therefore, miR-106a may achieve its anti-apoptotic effect by increasing the expression of these two proteins. At the same time, miR-106a can also enhance the production of IL-1β, IL-6, IL-8 and TGF- β, and thus affect the tumor microenvironment to contribute to the development of drug resistance.

Enhanced cell detoxification capacity is another important mechanism for the development of drug resistance [25]. GST-π is an important detoxifying enzyme in cells. In this study, we found that miR-106a positively regulates the expression of GST-π, indicating that miR-106a can reduce drug cytotoxicity by enhancing the detoxification capability of cells through GST-π.

ERCC1 is an important protein in the DNA repair pathway. ERCC1 expression is increased in tumor cells [26]. One mechanism of action of the anticancer drugs is to destroy the DNA integrity of the cells to activate the apoptosis pathway. In this study, we found that miR-106a increased the expression of ERCC1, indicating that miR-106a also plays an important role in the regulation of the DNA repair pathway. MiR-106a likely enhances tumor cell drug resistance by promoting the DNA repair pathway.

In addition, the results of this study indicate that miR-106a activates important oncogenic signaling pathways. MiR-106a can activate AKT and enhance the transcription of NF-κB, Twist, AP-1 and Snail. These are all important signals for triggering tumorigenesis and enhancing the anti-apoptotic capability of tumor cells [27].

In conclusion, this study revealed that miR-106a played an important role in the development of drug resistance to cisplatin and gefitinib in glioma. It can reduce the cytotoxicity of drugs and enhance drug resistance through the following mechanisms: (1) activating the oncogenic signaling pathway; (2) up-regulating RhoE and Topo-II; (3) enhancing the expression of CDX2 and up-regulating MDR1 and MRP1 to improve the drug efflux capability of the cells; (4) increasing the expression of GST-π to enhance the detoxification capability of the cells; (5) up-regulating ERCC1 to activate the DNA repair pathway; (6) promoting the production of cytokines relevant to drug resistance.
